# 'MiLES' population-based survey of the incidence and prevalence of systemic lupus erythematosus in Southeastern Michigan

**DOI:** 10.1186/ar3982

**Published:** 2012-09-27

**Authors:** EC Somers, W Marder, P Cagnoli, EE Lewis, P DeGuire, C Gordon, CG Helmick, L Wang, JJ Wing, JP Dhar, J Liesen, WJ McCune

## Background

We estimated the incidence and prevalence of systemic lupus erythematosus (SLE) in a sociodemographically diverse southeastern Michigan source population of 2.4 million.

## Methods

SLE cases fulfilling American College of Rheumatology (ACR)SLE classification criteria (primary case definition) or rheumatologist-judged SLE (secondary definition) and residing in Wayne or Washtenaw Counties during 2002 to 2004 were included. Case finding was performed from six source types, including hospitals and private specialists. Age-standardized rates were computed and capture-recapture performed to estimate under-ascertainment of cases.

## Results

Overall age-adjusted SLE incidence and prevalence per 100,000 were 5.5 (95% CI = 5.0 to 6.1) and 72.4 (95% CI = 70.4 to 74.4); capture-recapture adjusted estimates were 5.6 (95% CI = 5.1 to 6.2) and 71.8 (95% CI = 69.8 to 73.8). For all women the incidence was 9.3/100,000; prevalence was 128/100,000. SLE prevalence was 2.4-fold higher in blacks than whites, and 10-fold higher in women than men. Among the incident cases (ACR definition), mean age (± SD) at diagnosis overall was 39.2 ± 16.6 years. Blacks had a higher proportion of renal disease and end-stage renal disease (40.3% and 15.1%) versus whites (18.7% and 4.5%); blacks with renal disease were diagnosed with SLE at significantly younger age (33.9 ± 15.0 vs. whites 41.9 ± 21.3, *P *= 0.04).

